# Effect of estradiol on in vitro maturation of immature oocytes in cyclophosphamide-induced premature ovarian failure in NMRI mice: An experimental study

**DOI:** 10.18502/ijrm.v23i5.19263

**Published:** 2025-07-29

**Authors:** Yasaman Sohani, Mahnaz Azarnia, Hadis Zeinali, Sajed Khaledi, Mehdi Mehdinezhad Roshan

**Affiliations:** ^1^Department of Animal Sciences, Faculty of Biological Sciences, Kharazmi University, Tehran, Iran.; ^2^Department of Anatomical Sciences, Faculty of Medical Sciences, Tarbiat Modares University, Tehran, Iran.; ^3^Department of Reproductive Biology, Faculty of Advanced Medical Sciences, Tabriz University of Medical Sciences, Tabriz, Iran.

**Keywords:** In vitro egg maturation, Estradiol, Cyclophosphamide, Premature ovarian failure.

## Abstract

**Background:**

Premature ovarian failure (POF) is a condition characterized by the loss of ovarian function, leading to infertility.

**Objective:**

This study aims to examine how estradiol supplementation impacts the in vitro maturation of immature oocytes in NMRI female mice exhibiting cyclophosphamide-induced POF.

**Materials and Methods:**

In this experimental study, 15 female NMRI mice (8-10 wk, 30 ± 5 gr) were divided into 3 groups: control, sham, and treatment. The treatment group was divided into 3 categories. Treatment group 1, which added 0.5 μg/ml estradiol to the basic culture medium, and treatment groups 2 and 3, which were added to the culture medium, respectively. They added 1 and 1.5 µg/ml of estradiol to their basic culture medium. The treatment group received cyclophosphamide injections for 21 days to induce POF and then was treated with different doses of estradiol. The sham group also received all necessary interventions except estradiol treatment. After the induction, histological studies were conducted on the ovaries of all groups. Additionally, after stimulating and separating the ovules from the ovary, they were cultured in in vitro.

**Results:**

Analysis of oocyte maturity stages showed distinct features. Germinal vesicle oocytes' lowest percentage was in control (3.13%), and highest in sham (76.25%) (p < 0.0001). Estradiol dose inversely affected immature oocyte maturation, with the lowest in treatment group 3. Germinal vesicle breakdown oocyte percentage increased with estradiol dose. Metaphase II oocytes' highest maturation was in control (64.00%), and lowest in sham (5.00%) (p < 0.0001). Treatment groups showed varying rates. Degenerate oocyte percentages were lowest in control (1.80%), and highest in sham (10.27%) (p < 0.0001).

**Conclusion:**

Our study showed that using a dose of 1.5 μg/ml estradiol in vitro results in the highest oocyte maturation in the POF condition and contributes to the existing knowledge on POF and provides insights into potential therapeutic interventions aimed at improving fertility outcomes in individuals with POF.

## 1. Introduction

Premature ovarian failure (POF) is a condition characterized by the loss of ovarian function before the age of 40, leading to infertility and hormonal imbalances. The cause of POF is influenced by genetic, immunological, metabolic, viral, and pharmacological factors (1). One of the major causes of POF is exposure to chemotherapeutic agents, such as cyclophosphamide, which is commonly used in cancer treatment (2). While the detrimental effects of cyclophosphamide on ovarian function have been extensively studied, exploring potential strategies to mitigate its impact remains a significant area of research (3). The process of oocyte maturation plays a crucial role in fertility and successful reproduction. In recent years, in vitro maturation (IVM) has emerged as a promising technique for assisting the maturation of immature oocytes (4). By providing an alternative approach to traditional in vivo maturation, IVM offers the potential to overcome the limitations associated with POF and enhance fertility outcomes (5).

Estradiol, a key hormone in the estradiol-estradiol receptor signaling pathway, has been widely recognized for its role in follicular development and oocyte maturation. Studies have shown that estradiol supplementation during IVM can enhance oocyte quality and subsequent embryo development (6). However, the specific effects of estradiol on the IVM of immature oocytes in the context of POF caused by cyclophosphamide remain largely unexplored (7).

Therefore, the objective of this study was to investigate the effect of estradiol supplementation on the IVM of immature oocytes in NMRI female mice with POF induced by cyclophosphamide (8). By elucidating the impact of estradiol on oocyte maturation in this specific model, we aim to provide valuable insights into the potential therapeutic strategies for improving fertility outcomes in patients with POF (9).

The findings of this study could have important clinical implications, as they may contribute to the development of novel interventions to enhance oocyte maturation and improve the reproductive outcomes of individuals experiencing POF (10). Additionally, a better understanding of the underlying mechanisms involved in estradiol-mediated oocyte maturation could pave the way for personalized treatment approaches tailored to the specific needs of affected individuals (11).

In summary, this study aims to investigate the effect of estradiol on the IVM of immature oocytes in NMRI female mice with POF induced by cyclophosphamide. The results of this research have the potential to advance our understanding of the mechanisms underlying oocyte maturation and may offer new strategies for fertility preservation and management of POF.

## 2. Materials and Methods

### Laboratory animal

In this experimental study, 15 female NMRI mice (8–10 wk, 30 ± 5 gr) were selected for the study (purchased from Royan Institute, Tehran, Iran). The mice were randomly divided into 3 groups control, sham, and model (n = 5/each). The reason for choosing these animals was their genetic and physiological similarities to humans, as well as their ease of maintenance. Animals were maintained in the animal room at the Faculty of Biological Sciences, Kharazmi University, Tehran, Iran at 20–25 C with a 12-hr light/dark cycle.

Sufficient water and food were provided for the animals during the research period. To acclimate the animals to the new environment and eliminate stress caused by transportation, they were transferred to the housing facility 1 wk before conducting the experiments. The model group received cyclophosphamide at a dose of 20 mg/kg of body weight for 21 days via intraperitoneal (IP) injection to induce POF.

### Inducing POF using cyclophosphamide

To achieve this, the mice were first weighed, and based on their weight, cyclophosphamide (Baxter, Germany) was injected via IP for 21 days at a specific time using an insulin needle. During this period, the mice's weight changes were evaluated every 5 days to determine the dosage of cyclophosphamide injection and the likelihood of inducing failure in the mice (12).

### Histological studies

To confirm the induction of POF, after 21 days of inducing this condition using cyclophosphamide, the ovaries of control and model mice were extracted and fixed in Bouin's fixative. Then, tissue processing was performed using a tissue processor device. The samples were embedded in paraffin blocks and subsequently sectioned using a microtome. The sections were stained with hematoxylin-eosin and qualitatively examined.

### Preparation of tissue sections

Several steps are involved in preparing the samples for microscopic examination, including fixation, dehydration, paraffin infiltration, embedding, sectioning, staining, and mounting as described: 1) fixation: Bouin's solution was used for fixation, with a duration of 24 hr. 2) dehydration: different concentrations of alcohol were used in this process. 3) preparation for paraffin infiltration: after dehydration, the samples underwent paraffin infiltration, where paraffin was placed in the oven at 60 C. 4) impregnation and paraffin embedding: the samples were impregnated with 60% paraffin and were placed in paraffin for 2 hr in 2 separate intervals.

Then, the samples were embedded in metal molds using molten paraffin. After cooling and solidification of the paraffin, the prepared blocks were removed from the molds and stored in the refrigerator. 5) preparation of microscopic sections: after embedding, serial sections with a thickness of 6 microns were prepared from the samples using a microtome. To eliminate wrinkles, the sections were transferred to a water bath at 50 C, and then they were sequentially placed on albumin-coated slides and allowed to dry at room temperature. 6) staining: hematoxylin-eosin staining was used to study the slides under a light microscope. 7) mounting: after the above steps, the slides were mounted using Entellan mounting medium. Finally, the cell nuclei appeared purple, while the cytoplasm and extracellular matrix appeared pink.

### Preparation of culture medium and droplet placement

Eagle's minimum essential medium α (MEMα) culture medium was purchased from a company called (Pishgam Paya Zist, IR). The medium was prepared for oocyte culture by adding additional compounds based on the study groups. Each vial of human chorionic gonadotropin (Arman Pharmed Daru, Iran) contained 5000 IU of human chorionic gonadotropin powder, and each vial of follicle-stimulating hormone (FSH, Cinagen, IR) contained 7.5 IU of FSH. Hormones were dissolved in the culture medium to have 1 international unit of hormone per 100 μ l, and then the resulting solution was aliquoted into microtubes. When using each microtube, it was diluted with 1 ml of MEMα. After preparing the culture medium, 100 μ l droplets were evenly placed in the culture dish using a pipette and were immediately covered with mineral oil. This was done to prevent droplet evaporation, potential contamination, and to maintain the pH balance. In this stage, to equilibrate the droplets' temperature and gas concentration with the incubator, the culture dishes containing the droplets were placed in the incubator for 12–16 hr (13).

### Oocyte stimulation, oocyte collection, and oocyte retrieval

Ovaries were located at the end of each uterine horn. Following careful tracing of uterine horns using fine forceps, the uterine tube was identified. Surrounding fat tissues were eliminated to facilitate separation. The junction between the uterine tube and ovary was meticulously cut with scissors, freeing the ovaries. The ovaries were immediately placed in a Petri dish with a culture medium. For better visualization and oocyte retrieval, the dish was positioned under a stereomicroscope. Gently bringing the ovaries into the dish's depth, they were held near the swollen oviduct region. A careful puncture with an insulin needle released oocytes. Oocytes were collected (at least 10 were extracted in each group) and transferred to pre-prepared culture medium droplets using a pipette, and then incubated for 24 hr. Maturation outcomes were subsequently observed.

Different stages of oocyte maturation were investigated. These steps include the following:

1. Oocyte stimulation and euthanasia: oocyte stimulation was initiated through the administration of 7.5 IU of pregnant mare serum gonadotropin (Hypra Co., Spain) via IP injection. After 46–48 hr, mice were euthanized using cervical dislocation, ensuring humane handling.

2. Ovary extraction and preparation: following euthanasia, the mice were sterilized with 70% ethanol and positioned on a dissecting tray. The abdominal skin was delicately opened to access the uterine horns, allowing for careful extraction of the ovaries. Fat tissues around the ovaries were removed to facilitate subsequent steps.

3. Ovary transfer and dissection: the ovaries were placed in 200 μ l culture medium (MEMα) droplets with 5% fetal bovine serum. Excess fat was removed, and the ovaries were then moved to separate droplets (300 μ l) for dissection under an inverted microscope. The goal was to release immature oocytes at the GV stage, either with or without cumulus cells.

4. Cumulus cell separation: cumulus cell complexes were isolated by pipetting, carefully separating them from the oocytes. This step aimed to analyze the oocyte maturation process independent of cumulus cell influence.

5. Oocyte selection and IVM: immature oocytes with distinct features, clear cytoplasm and appropriate perivitelline space, were meticulously chosen under an inverted microscope for IVM. The selected oocytes from each group were then cultured in controlled conditions for 24 hr.

6. Incubation and examination: the selected oocytes were cultured in a medium-covered droplet environment under controlled conditions with 5% carbon dioxide and maintained at 37 C. After the 24-hr incubation period, the maturation stages of all groups were meticulously examined and documented using an inverted microscope.

### Ethical Considerations

The animal experiments were approved by the Ethical Committee of Kharazmi University, Tehran, Iran (Code: IR.KHU.REC.1401.034). All methods were carried out following relevant guidelines and regulations. Also, all methods are reported according to the ARRIVE guidelines.

### Statistical Analysis 

First, all data were analyzed for normality of their distribution using Kolmogorov-Smirnov analysis. Then, due to the normality of the data, the one-way ANOVA method and Dunn post hoc test were used to compare the groups. Data were reported as mean ± SD, with p < 0.05 indicating significance. SPSS software (IBM, SPSS Statistics, version 27) was used to analyze the data and Graph Pad Prism software (version 10.1.0) was used to draw the graphs.

## 3. Results

**Figure 1:**
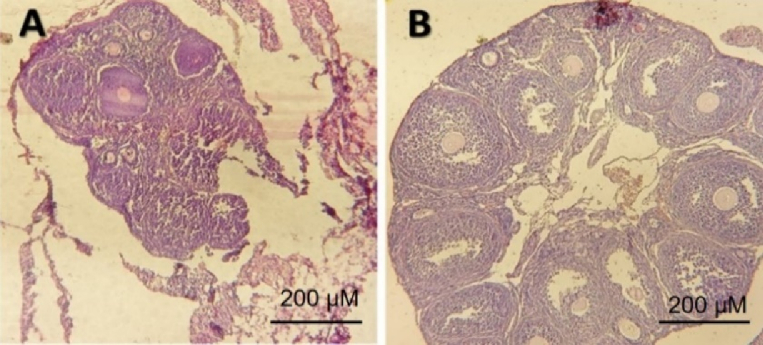
Mouse ovarian tissue in A) POF model, B) Control group (x10 magnification).

The results of our study comparing the control group with the model group of POF induced by cyclophosphamide revealed significant follicular destruction and damage to the ovarian tissue in the POF model group. These findings provide evidence for the successful induction of POF using cyclophosphamide as a modeling drug, as supported by previous research that highlighted the profound effects of chemotherapy drugs on ovarian tissue and follicular destruction (Figure 1). The extent of follicular destruction observed in our model group underscores the relevance and validity of using cyclophosphamide to induce POF in our study.

**Figure 2:**
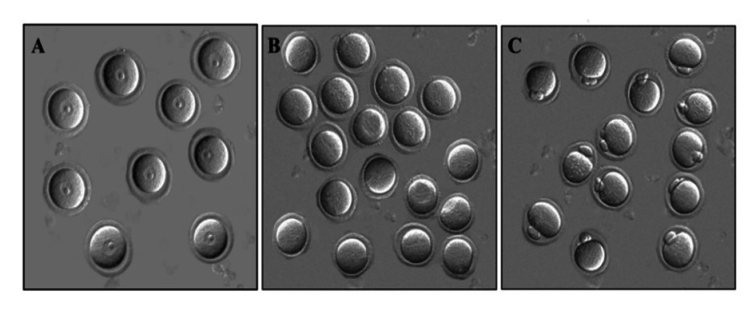
Oocytes at different stages of maturity: A) GV, B) GVBD, and C) MII (x200 magnification). GV: Germinal vesicle, GVBD: Germinal vesicle breakdown, MII: Metaphase II.

Regarding the examination of oocytes in different stages of maturity, our observations included germinal vesicle (GV), germinal vesicle breakdown (GVBD), metaphase II (MII), and degenerate oocytes. GV oocytes exhibited distinct features, such as clear cytoplasm, a region of zona pellucida, and dense cumulus cells. GVBD oocytes represented the stage at which meiosis resumes, with the nuclear membrane being broken down. MII oocytes were characterized by the presence of the first polar body and indicated complete maturation. In contrast, degenerate oocytes represented fragmented or damaged oocytes within the zona pellucida (Figure 2).

**Figure 3:**
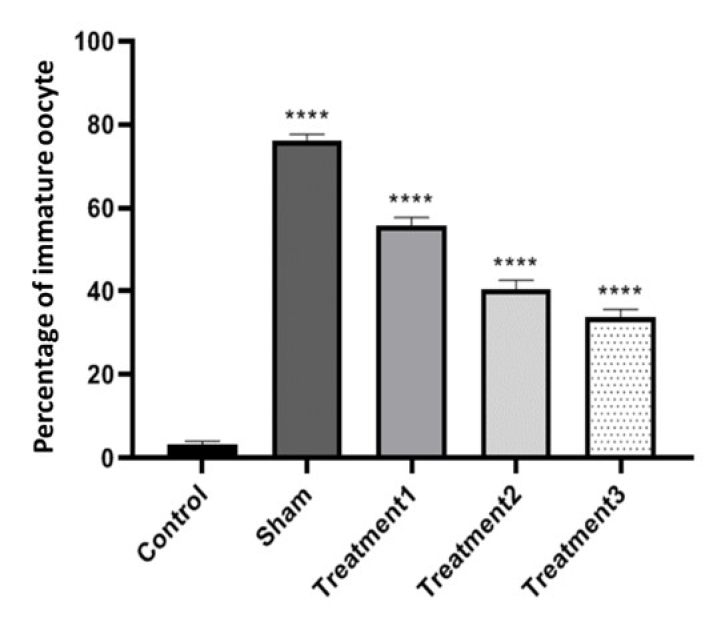
Comparison of the percentage of immature oocytes after culture in the culture medium for the control group, sham group, and treatment groups. ****P < 0.0001.

When examining the percentage of GV oocytes, we observed the lowest percentage in the control group (3.13 ± 0.78) and the highest in the sham group (76.25 ± 1.28). Increasing the dose of estradiol in the maturation environment resulted in a decrease in the maturation percentage of immature oocytes, with the lowest rate observed in treatment group 3 (maturation medium containing 1.5 μ g/ml estradiol). The differences between all groups were found to be significant (p < 0.0001) (Figure 3).

**Figure 4:**
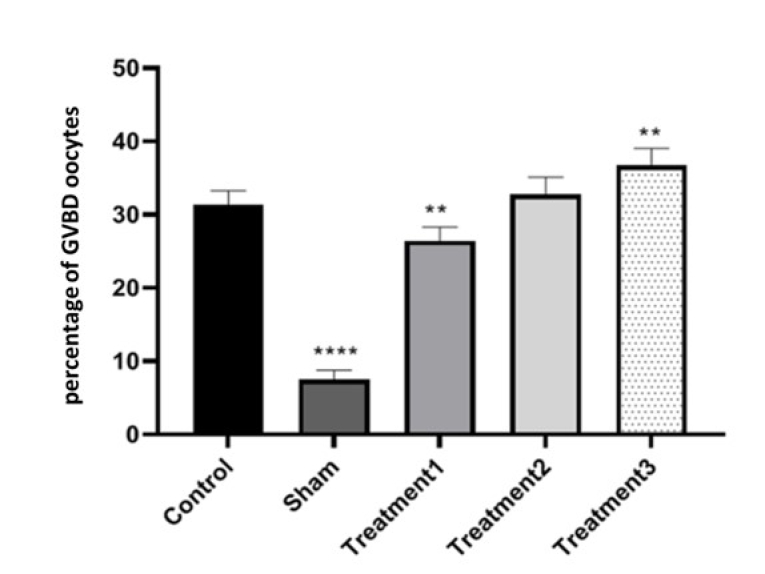
Comparison of the percentage of germinal vesicle breakdown (GVBD) oocytes after culture in the culture medium (containing different doses of estradiol) for the control group, sham group, and treatment groups: treatment 1, treatment 2, treatment 3. **P < 0.01, ****P < 0.0001.

In terms of the percentage of GVBD oocytes, the lowest percentage was observed in the sham group (7.25 ± 1.26), while the highest was observed in treatment group 3 (maturation medium containing 1.5 μ g/ml estradiol) (36.25 ± 2.37). The results indicated that the percentage of GVBD oocytes increased with the dose of estradiol in the maturation environment. The differences between the control and treatment groups were not significant, but significant differences were observed among the other groups at a level of p < 0.05 (Figure 4).

**Figure 5:**
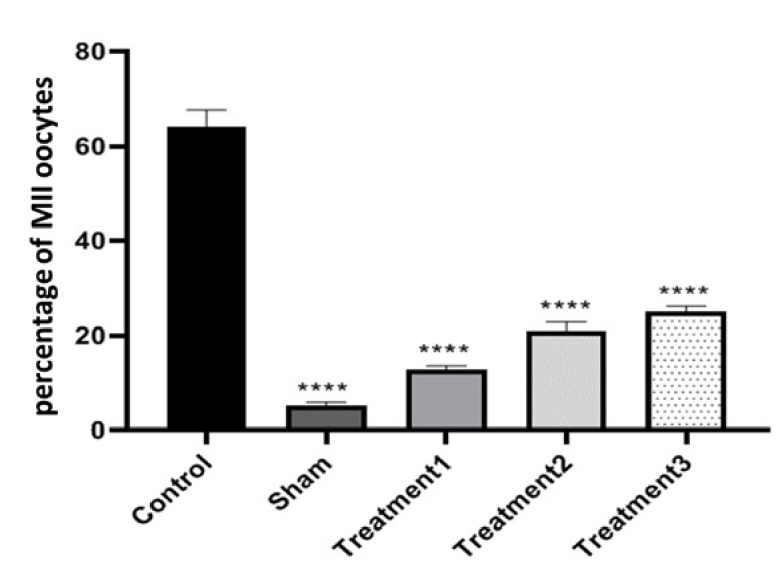
Comparison of the percentage of MII oocytes after culture in the culture medium (containing different doses of estradiol) for the control group, sham group, and treatment groups. ****P < 0.0001.

The percentage of MII oocytes showed the highest maturation rate in the control group (64.00 ± 0.85), whereas the lowest rate was observed in the sham group (5.00 ± 2.37). The 3 treatment groups with increasing doses of estradiol exhibited maturation rates of 12.70 ± 0.86, 21.00 ± 1.77, and 25.00 ± 1.12, respectively. The percentage of MII oocytes increased with the dose of estradiol in the maturation environment, similar to GVBD oocytes. The differences between all groups were significant at a level of p < 0.0001, and the difference between treatment groups 2 and 3 was significant at a level of p < 0.05 (Figure 5).

**Figure 6:**
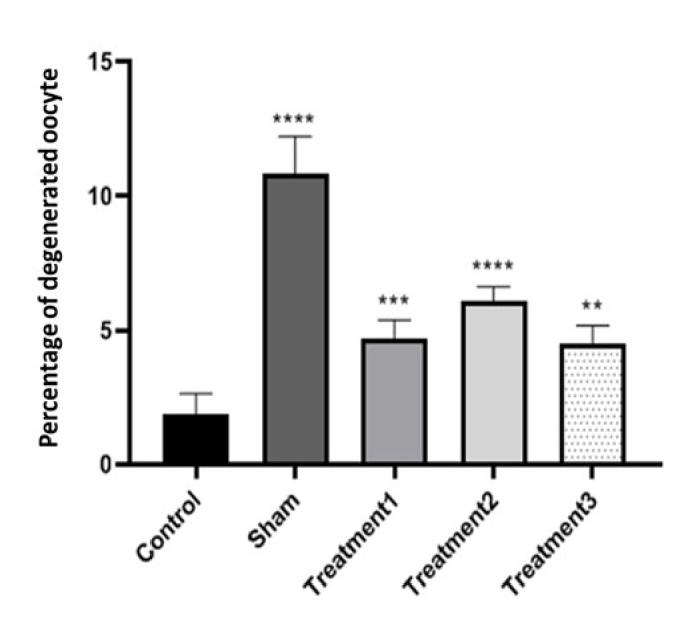
Comparison of the percentage of degenerated oocytes after culture in the culture medium (containing different doses of estradiol) for the control group, sham group, and treatment groups. **P < 0.01, ***P < 0.001, ****P < 0.0001.

**Figure 7:**
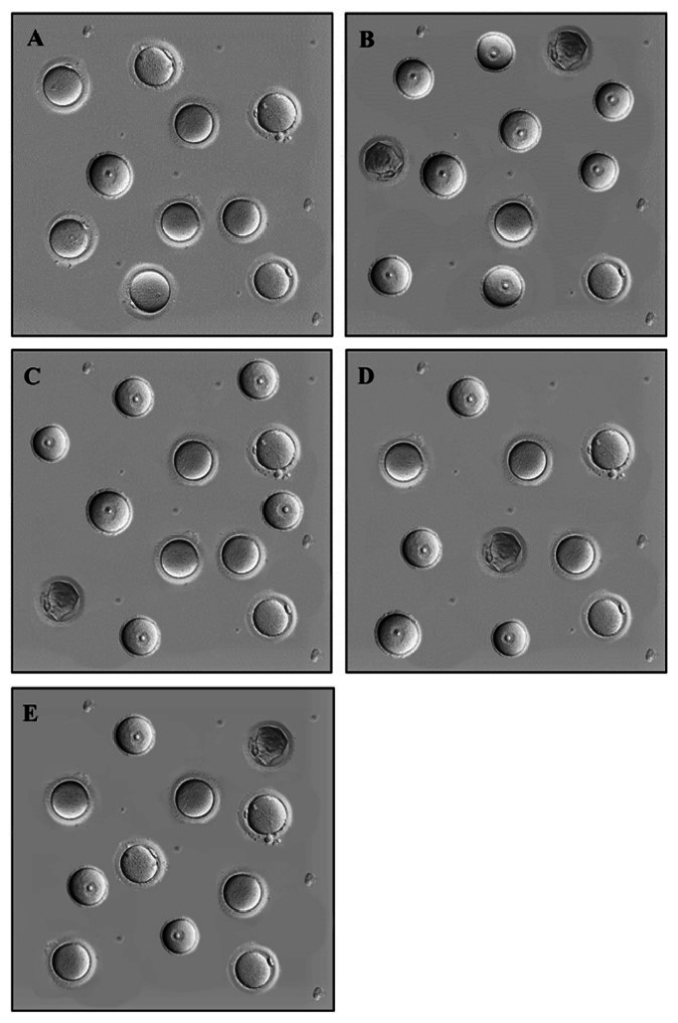
Comparison of oocyte maturation stages A) Control, B) Sham, C) Treatment 1, D) Treatment 2, and E) Treatment 3 (x100 magnification).

Analyzing the percentage of degenerate oocytes, we observed that the control group had the lowest percentage (1.80 ± 0.72), while the sham group had the highest percentage (10.27 ± 1.82). The treatment groups showed percentages of 4.58 ± 0.68, and 5.92 ± 0.76 for treatment 1 (maturation medium containing 0.5 μ g/ml estradiol), treatment 2 (maturation medium containing 1 μ g/ml estradiol), and treatment 3 (maturation medium containing 1.5 μ g/ml estradiol), respectively. As expected, the sham group exhibited the highest proportion of degenerated oocytes. The difference between the control and sham groups was significant (p < 0.0001), while differences between the treatment groups were also significant, indicating varying effects of estradiol on degenerate oocyte formation (Figures 6 and 7).

## 4. Discussion

The outcomes of our research comparing the control group to the model group, which experienced POF induced by cyclophosphamide, demonstrated notable follicular damage and ovarian tissue impairment in the POF model group. These findings provide evidence supporting the successful induction of POF through the use of cyclophosphamide as a modeling drug, consistent with previous studies highlighting the profound impact of chemotherapy drugs on ovarian tissue and follicular destruction. The extent of follicular destruction observed in our model group emphasizes the relevance and validity of utilizing cyclophosphamide to induce POF in our study.

There are a couple of studies that investigated the effect of estradiol supplementation on oocyte quality in women with POF and have demonstrated that estradiol administration improved oocyte developmental competence, mitochondrial function, and spindle formation, suggesting a positive impact on oocyte quality and potential for successful fertilization. These results highlight the potential of estradiol as a therapeutic agent to mitigate ovarian damage in POF (14–18).

Recently, a comparative investigation compared the effectiveness of estradiol and FSH for IVM of immature oocytes in a rat model of POF. This study evaluated the maturation rates, morphological features, and developmental competence of oocytes treated with estradiol or FSH, and results contributed to the understanding of the differential effects of these hormonal interventions on oocyte maturation in POF (19). Several studies have indicated that estradiol prevents glucocorticoid apoptosis in mice and enhances cell cycle progression via modulating the production of B-cell lymphoma 2, cyclin D, and cyclin E (20, 21). In the evaluation of GV oocyte percentages, the control group displayed the least proportion, whereas the sham group demonstrated the highest. Elevating the estradiol dosage in the maturation milieu resulted in a reduction in the maturation rate of immature oocytes, with the lowest rate observed within treatment group 3. Statistical analysis revealed significant variations among all groups. Furthermore, numerous investigations have delved into the molecular mechanisms governing oocyte maturation through estradiol in cases of POF. Consequently, directing attention toward the signaling pathways and alterations in gene expression prompted by estradiol treatment, and comprehending their influence on oocyte quality, facilitates the comprehension of tailored strategies to augment oocyte maturation in instances of POF (22–25).

Concerning the proportion of GVBD oocytes, the sham group exhibited the lowest percentage, whereas the highest proportion was evident in treatment group 3. The findings revealed a positive correlation between the estradiol dosage in the maturation setting and the percentage of GVBD oocytes. Although the disparities between the control and treatment groups lacked significance, noteworthy variations emerged among the remaining groups. Studies have shown that estradiol can decrease the level of reactive oxygen species, which can be beneficial for in vitro oocyte maturation. In one study, it was demonstrated that 17β-estradiol increased the nuclear maturation of porcine oocytes in a time-dependent manner, indicating that 17β-estradiol autophagy regulates cumulus-oocyte complexes during IVM. This effect is mediated through the phosphorylation of Cx43, which is regulated via the MEK/ERK signaling pathway (26, 27). A research has also shown that estrogen can modulate Ca^2+^ levels via the GPER-EPAC1 pathway and increase the production of SIRT1, which improves oocyte mitochondrial function throughout maturation (28). The control group displayed the most substantial maturation rate for MII oocytes, whereas the lowest rate was recorded in the sham group. Among the 3 treatment groups, characterized by escalating estradiol doses, the percentage of MII oocytes demonstrated an incremental trend in tandem with the estradiol dose in the maturation milieu, mirroring the pattern observed for GVBD oocytes. All group differences exhibited statistical significance, with a particularly significant contrast evident between treatment groups 2 and 3.

It has been demonstrated that the combination of progesterone and 17β-estradiol had a positive effect onthe cytoplasmic maturation of prepubertal and pre-ovulatory oocytes in monkeys. Within the body, oocyte growth and maturation are directly regulated by ovarian factors such as steroids, cytokines, etc. Among these factors, estradiol plays a significant role in oocyte maturation, follicular growth, ovulation, and in fertilization. This study provided the first evidence that estradiol and progesterone significantly improve the cytoplasmic maturation of mammalian oocytes (27). However, in another study conducted on frogs, it was shown that estradiol inhibits oocyte maturation (29).

On examining the proportion of degenerate oocytes, it was noted that the control group exhibited the minimal percentage, while the sham group displayed the maximum percentage. As anticipated, the sham group demonstrated the highest rate of degenerated oocytes. The contrast between the control and sham groups yielded statistical significance, and noteworthy variations were also observed among the treatment groups, highlighting divergent impacts of estradiol on the formation of degenerate oocytes. Overall, our findings highlight the significant impact of cyclophosphamide-induced POF on follicular destruction and histological changes in ovarian tissue. Furthermore, our study provides insights into the effects of estradiol supplementation on the maturation of immature oocytes. The varying percentages of GV, GVBD, MII, and degenerate oocytes across the control, sham, and treatment groups indicate the influence of estradiol on oocyte maturation.

### Strengths and limitations

Due to resource limitations, complementary cellular and molecular tests such as polymerase chain reaction and immunoassay were not used in this study. Therefore, further studies are needed to determine the role of estradiol in the process of egg maturation in vitro and its relationship with human egg maturation.

## 5. Conclusion 

These findings suggest that the use of an optimal dose of estradiol to mature immature oocytes in the setting of POF may be effective. The results of this study may also contribute to a better understanding of the mechanisms involved in oocyte maturation and may have implications for the development of strategies to enhance reproductive outcomes in patients with POF. It is important to note that further research is needed to elucidate the molecular pathways and mechanisms by which estradiol affects oocyte maturation in the context of POF. In addition, evaluation of other parameters such as hormone levels, gene expression patterns, and embryonic growth potential will provide a more comprehensive understanding of the effects of estradiol supplementation on oocyte maturation.

##  Data Availability

The data sets analyzed for the current study are available from the corresponding author upon reasonable request.

##  Author Contributions

M. Azarnia: Had full access to all of the data in the study and takes responsibility for the integrity of the data and the accuracy of the data analysis. M. Mehdinezhad Roshan and Y. Sohani: Article writing, editing, and data analysis. H. Zeinali and S. Khaledi: Monitored, evaluated, and analyzed the result of the study. All authors approved the final manuscript and take responsibility for the integrity of the data.

##  Conflict of Interest

The authors declare that they have no competing interest.
